# Markets, breastfeeding and trade in mothers’ milk

**DOI:** 10.1186/s13006-015-0034-9

**Published:** 2015-02-23

**Authors:** Julie P Smith

**Affiliations:** Australian National University, Canberra, Australia

**Keywords:** Breast feeding/economics, Infant formula, Formula feeding, Cost-benefit analysis, Cost of illness, Health priorities, Health promotion/economics, Child health, Infant mortality, National health programs/economics, Maternity leave, Maternal employment

## Abstract

This introduction to a special issue on the economics of breastfeeding draws attention to the lack of economic justice for women.

Human milk is being bought and sold. Commodifying and marketing human milk and breastfeeding risk reinforcing social and gender economic inequities. Yet there are potential benefits for breastfeeding, and some of the world’s poorest women might profit. How can we improve on the present situation where everyone except the woman who donates her milk benefits?

Breastfeeding is a global food production system with unsurpassed capacity to promote children’s food security and maternal and child health, but it is side-lined by trade negotiators who seek instead to expand world markets for cow’s milk-based formula. Regulators focus on potential risks of feeding donated human milk, rather than on health risks of exposing infants and young children to highly processed bovine milk. Similarly, policymakers aspire to provide universal health care access that may be unaffordable when two thirds of the world’s children are not optimally nourished in infancy, resulting in a global double burden of infectious and chronic disease. Universal breastfeeding requires greater commitment of resources, but such investment remains lacking despite the cost effectiveness of breastfeeding protection, support and promotion in and beyond health services. Women invest substantially in breastfeeding but current policy - epitomised by the G20 approach to the ‘gender gap’ - fails to acknowledge the economic value of this unpaid care work. Economic incentives for mothers to optimally breastfeed are dwarfed by health system and commercial incentives promoting formula feeding and by government fiscal policies which ignore the resulting economic costs.

‘The market’ fails to protect breastfeeding, because market prices give the wrong signals. An economic approach to the problem of premature weaning from optimal breastfeeding may help prioritise global maternity protection as the foundation for sustainable development of human capital and labour productivity. It would remove fiscal subsidies for breast milk substitutes, tax their sale to recoup health system costs, and penalise their free supply, promotion and distribution. By removing widespread incentives for premature weaning, the resources would be available for the world to invest more in breastfeeding.

## Background

It was recently reported that a major US retailer was considering selling human milk [1]. Though it turned out to be a hoax, the news story highlights the increasing relevance of market economics to feeding infants and young children, and points to some challenging issues policymakers must tackle.

Unregulated commodification and marketization of human milk risk reinforcing current social and gender economic inequities. Related policy and regulation need to improve a situation ‘where everyone except the woman who donates her milk benefits’ ([2], p 66).

### Breastfeeding, and the global spread of markets

Women’s ability to decide to breastfeed and to practice it optimally is increasingly affected by globalisation of food and food marketing systems, and greater maternal participation in labour markets through paid employment without workplace accommodation for the maternal role of women. As markets spread into new areas of human life, economic incentives, rather than traditional practice and mammalian biology, exert greater influence, including on child-feeding practices; especially, the market share of breastmilk substitutes expands.

Trade is conventionally seen as the lynchpin of global food security, and world trade in infant and young child milk formula and baby food is booming in emerging economies in Asia and Latin America. Global sales of baby foods - mainly bovine milk-based formulas - rose from US$18 billion in 1999 to US$58 billion in 2013, and are forecast to rise to US$89 billion by 2017 [3]. Yet it is wrong to assume that the current international trade and investment framework will improve food security for infants and young children, as it fails to prioritise optimal breastfeeding, public health, and the human rights of mothers and children [4]. Breastfeeding is itself a global ‘food system’ with unsurpassed capacity to promote food security and health for infants and young children; yet it is too frequently side-lined by trade negotiators chasing national competitive advantage in selling milk formula.Trade in milk formula also fails to optimise human development due to the significant cognitive development disadvantage of babies fed infant formula before six months compared to those exclusively breastfed [5]; the deficit from this early life exposure to suboptimal nutrition is akin to low level pre-natal lead exposure in its effects on countries’ educational achievement [6], yet effects on the population’s dietary quality – and on future chronic disease and labour productivity – are increasingly left out of consideration in trade agreements [7].

The paper by Libby Salmon in this special issue identifies the public policy conflicts between international trade and optimal breastfeeding promotion and shows how more integrated and balanced policies could result from applying a food security analytical framework to the challenge of ensuring optimal infant and young child nutrition [8]. For example, food security encompasses accessibility. For infants and young children, this means accessing human milk and breastfeeding. Can working mothers and their infants access breastfeeding if paid maternity leave is not available or if work arrangements serve as a barrier to breastfeeding?

Likewise, should health care systems fail to finance ‘adequate nutrition’ for infants but rely instead solely on the generosity of mothers? In some countries, human milk is available for all medically vulnerable infants via publicly funded hospital milk banks [9,10]. In other countries, privately insured mothers may purchase donated human milk accessed through milk banks, while the infants of the uninsured may miss out on their mammalian birth right. Governments provide fiscal and regulatory incentives to make therapeutic human blood products widely and safely available, yet few health systems encourage institutions to provide human milk for sick or hospitalised infants To date, the main regulatory policy responses to the various forms of trade in human milk focus on warning of potential risks of transferring harmful bacteria or virus or illicit drugs or medications through donor milk given to vulnerable infants [11]. Strong public condemnation by health authorities of milk sharing and trading prompts the question of whether there is a double standard, even conflicts of interest; why are the comparable health risks of feeding infants and young children bovine–based milk formula not also consistently and vocally highlighted by the authorities, and why are mothers considered incompetent to make informed choices to purchase human milk [12]? George Kent’s paper on the global regulation of infant formula argues that regulators fail to require adequate information on the risks of exposure to commercial milk formulas [13], and challenges them to properly justify the unsubstantiated presumption that these formulas are ‘safe’ and provide ‘adequate nutrition’ as the sole food for infants. When human milk is an accessible alternative, by what standard is infant formula judged nutritionally ‘adequate’ by food safety regulators?

### Selling mothers’ milk

The growing commodification of human milk and breastfeeding creates complex and controversial policy problems in eliciting and allocating its supply. For the past century, the operating business model for human milk sharing has been the ‘gift economy’, with the mother who provides the milk being unremunerated [14,15]. However, technological change is facilitating the trading and exchange of human milk and employment in wet-nursing [2,16-20], with human milk now collected, processed and sold as a commercial enterprise [21].

Whether these niche markets in human milk widen or, alternatively, discourage infants’ and mothers’ access to breastfeeding depends partly on the social and institutional context, and on how health regulatory policy addresses the market failures and inequities associated with the current incentives for formula feeding [22]. For example, as well as making human milk available to more babies, greater trading and exchange of human milk could mean greater societal recognition of the economic value of breastfeeding, and might enable human milk or breastfeeding to better compete with bovine milk-based substitutes. Indeed, women – as sole producers of this uniquely valuable ‘health food’ - can earn a decent livelihood as wet-nurses [23], or supplement their often meagre income by selling their surplus milk through the newly emerging markets and online trading systems [16].

On the other hand, markets for human milk could simply mean new avenues for the rich and strong to exploit the poor and vulnerable, while reducing some women’s ability and incentive to commit to optimal breastfeeding their own infants. For example, a report of a reluctant surrogate mother in India who was required to be a wet nurse and nanny under contract to a Canadian couple illustrates the potential unequal power dynamics and patriarchal or social class structures that already underpin markets in ‘reproductive services’ [24]. How might national authorities respond to the international trafficking of women coerced into providing reproductive services such as wet-nursing? One wonders also, to what extent is consumer demand for human milk and wet-nurses underpinned by the lack of adequate maternity leave to enable optimal breastfeeding in countries such as the US or China?

### Breastfeeding minimises health care costs and enhances equity, but who pays for the free lunch?

Well known for preventing infectious illness in infants, young children and protecting the health of their mothers, breastfeeding also promotes development of the immune system and cognitive development, and influences later life chronic disease risk, and contributes to maternal reproductive health in many ways [5,25-29]. Even where geographically or socially disadvantaged populations are poorly served by health services, breastfeeding is a universally accessible ‘health service’ and a tool for equity in child health [30], and can promote rights to reproductive health for even the poorest women, including through adequate birth spacing [31].

There are calls worldwide for universal access to healthcare services (such as by the Lancet Global Health Commission [32]). However, the recent dramatic decline in breastfeeding in Asian Pacific countries, notably in China [4], poses the question of whether the world can afford universal health care without first protecting and promoting universal breastfeeding. Based on estimates of the costs of artificial infant feeding in developed countries [33-41], health care systems in low- and middle-income countries will carry a heavy double burden of infectious illness and chronic disease if breastfeeding is not urgently protected from the ‘white gold rush’ of formula sales underway in the Asia Pacific region.

However, universal support for women to succeed in breastfeeding requires investment, to address the considerable incentives against it. Although support for breastfeeding is widely acknowledged to be one of the most cost-effective ‘interventions’ [42], it is one of the most underfunded. The resourcing and implementation of the WHO/UNICEF *Global Strategy for Infant and Young Child Feeding* remains weak at the global and country level despite lip service paid to its value in international and national policy statements [43]. The paper by Holla and colleagues in this issue includes a tool estimating the cost of universal implementation of the *Global Strategy* [44]. This research reinforces awareness that investing in breastfeeding also directs resources to the world’s poorest women, and helps address gender equity, while contributing to a sustainable food, nutrition and health system for the world’s most vulnerable infants and young children.

### Incentives for breastfeeding

There is a growing interest in offering financial incentives to motivate health behaviour change. We can learn much from companies about how to market effectively to mothers [45]. Formula manufacturers are very successful in using ‘incentives’ to motivate, and connect with mothers. It has been posited that as a social marketing technique, financial incentives may be relevant to increasing breastfeeding [46].

From an economic perspective though, the critical issue is the less visible incentives, for or against breastfeeding, operating in health care systems and institutions, and created by labour markets and employment arrangements. While most such research has focused on health care impact on mothers’ behaviours [47-49], and the unintended consequences [50,48], the central question is, what incentives for breastfeeding protection, support and promotion are faced by the other stakeholders in infant and young child feeding, including formula companies, governments, and health care providers?

Market mechanisms fail to protect breastfeeding because of the distorted incentive structures that influence infant feeding decisions, and the corresponding lack of a commercially motivated ‘Mothers’ Milk Incorporated’ to protect, promote and support breastfeeding [22]. The paper by Elien Rouw and colleagues [51] well illustrates the perverse economic incentives that operate for mothers, hospitals and health care professionals in Germany, who face significant financial penalties for breastfeeding or its protection, support or promotion. Public policy on the other hand, may neglect breastfeeding because its economic worth is rarely measured in economic terms [52], and because women’s economic contribution through time spent in breastfeeding and other unpaid care work is often discounted and ignored, and, therefore, invisible [53].

The likely harm to breastfeeding from unregulated marketing and promotion and use of breast milk substitutes by government, health care institutions and industry is recognised at the international level in the existence of the *International Code* of *Marketing of Breast-milk Substitutes* [54] and the *Codex Code of Ethics for International Trade* in Food [55]. Yet, within countries, the International Code is inconsistently applied, and rarely encompasses export industries, so companies have little incentive to behave ethically [56]. Currently, the regulation of marketing in developed countries and a trend to return to breastfeeding, is giving formula companies an incentive to shift their attention to developing countries in the Asia Pacific region where regulatory and health care systems are embryonic or weak, and maternity protection minimal. Women are being harnessed into formal employment by G20 policy priorities to address ‘the gender gap’ in labour force participation [57]. As mothers of young children also carry an unequal burden of unpaid care work [58], time pressed women concentrated in poorly paid jobs and with unsupportive work conditions are particularly vulnerable to aggressive marketing of infant and toddler milk formulas [59]. Boosting the market economy by creating incentives which shrink the unpaid economy is not economic progress [60].

The fiscal policies of all governments are strongly concerned with influencing incentives and disincentives, and tobacco and alcohol taxes are well known to affect health behaviour [61]. Governments and international agencies heavily subsidise vaccination programs to give families incentives to immunise their children [51]. Breastfeeding contributes to maternal and child health across a much wider sphere. So what incentives do governments create for breastfeeding through their fiscal policies?

Surprisingly, government fiscal policies worldwide provide incentives for families to use formula rather than breastfeed optimally. For example, formula use is heavily subsidised by governments through community welfare programs [62]. In the US, the Special Supplemental Nutrition Program for Women, Infants, and Children (WIC) program purchases more than half of all formula sold, potentially underwriting the price of formula worldwide [3,63]. In Australia, infant milk formula is exempt from the national goods and services tax, yet lactation aids such as breast pumps are taxed at 10%. In China, formula sales are promoted by government subsidies which aim to develop the domestic industry and to encourage research into new formula products [64,65]. Labbok and colleagues have noted in this journal [66] that advocacy for a Breastfeeding Budget was a key policy action for financing gender equality at the 2008 UN Commission on the Status of Women [67]. Yet government-supported breastfeeding programs remain tiny compared to the approximate 10% of sales spent by the industry on marketing baby food products [68].

The availability of free formula is surely a financial disincentive to protect, promote and support breastfeeding. Incentives for formula feeding are also intrinsic to health financing arrangements which allow training and education for health workers to be provided by formula companies. Around 60 per cent of maternity services globally are not Baby Friendly Hospital Initiative (BFHI) designated [62]. Such hospitals provide free formula as a matter of course for new-borns [64,65], yet can require mothers to purchase their own breast pump in order to continue expressing and giving milk for their hospitalised infants. Hospital decision-makers perceive BFHI accreditation, which simply provides quality of care [12], as expensive even though higher breastfeeding rates reduce health care costs [33-38]. One needs to look no further than China in 2013 [69-72] or Italy in 2014 [73] to find system-wide incentives for maternal and child health care systems and health professionals to promote formula, and hence, create barriers to breastfeeding success. While health systems and workers worldwide accept gifts, training and education opportunities, and free product from the baby food industry, in contravention of the International Code, how can any token incentives for breastfeeding possibly work?

The strong commercial motivations for the promotion and advocacy of formula feeding by companies are clear, but it would seem that government policies, health systems and health workers reinforce, rather than redress, the commercial incentives. As Finch and colleagues ([74], p. 982) observed with considerable understatement in 2002, ‘improving rates of breastfeeding when formula is free is a great challenge.’

Most importantly though, consideration of financial incentives must also address the economic issue of women’s opportunity cost of time, and relatedly, the employment and workplace barriers to breastfeeding facing many mothers around the world. Breastfeeding is not, contrary to the rhetoric, a free good [75,76]. It has long been recognised that women’s time and skill are essential for optimal breastfeeding [75], and this has been shown by time use research in Australia to be economically costly for women [77]. However, most cost-benefit studies of breastfeeding, or initiatives to promote it, fail to acknowledge the maternal time costs of breastfeeding and the costs of breastfeeding support, as such studies are usually conducted from a narrow health system or health provider perspective. For the right incentives to operate in regard to feeding infants and young children, women’s unpaid care work must be adequately valued and resourced, and adequate paid maternity leave and supportive employment provided for all working women. The feminist ‘silence on suckling’ [78] needs to be redressed by feminist economic analysis of the economic value of women’s reproductive work including breastfeeding, and strong advocacy of maternity protection not only in the G20 countries but globally. Improving rates of breastfeeding when the economic burden of lost earnings and career opportunities falls heavily on the poorest women is as much of a challenge as improving breastfeeding when formula is free.

### Researching economic aspects of breastfeeding

An economic approach to the problem of premature weaning from optimal breastfeeding would start by removing both the explicit and implicit fiscal subsidies for the marketing, promotion and use of breast milk substitutes. The International Code would be enforced throughout the health system to ban provision of free or subsidised supplies, and prevent industry from conducting health worker training and education in formula feeding. Health care services and hospitals could be rewarded for BFHI accreditation with additional funding to achieve a more appropriate alignment of the health system costs and rewards of breastfeeding protection, promotion and support. An excise tax on sales of breast milk substitutes could be designed to recoup the health costs of premature weaning. Combined with enforcement of the International Code, this would help correct the market failures that undermine health institutional incentives to support breastfeeding. Revenue could be used to address health system barriers to breastfeeding support. Reflecting breastfeeding’s long-term contribution to health and human capital building, enhanced access to paid maternity leave for mothers of new-borns might be funded through appropriate combinations of general income tax, social security or social insurance fund contributions and payroll levies, and through an international fund created to assist countries to implement adequate maternity protection throughout the world. This approach to financing maternity protection would share the economic costs of reproduction with wider society, and minimise incentives for employers to dismiss or not hire women of child-bearing age. Such approaches are controversial, but UNICEF data show optimal breastfeeding rates to have been virtually stagnant for decades. Indeed, industry data shows rapid expansion of milk formula sales, and infers very substantial global declines in breastfeeding [4].

## Conclusion

Research evidence and scholarly debate on the economic aspects of breastfeeding are urgently needed to ensure that the public policy and regulatory discussion has a strong conceptual framework and evidence base, and that policy formulation is not dominated and distorted by powerful sectional interests.

Breastfeeding and expressed human milk may be given freely by women everywhere, but that is no reason for it to be considered of no economic value, and why women should not profit from it. Marketisation of human milk and breastfeeding should not reinforce and validate the unrequited burdens and unequal care responsibilities of the world’s long-suffering and overloaded mothers.

A sustained research focus on the economic aspects of breastfeeding would highlight the key economic factors and incentives that shape mothers’ decisions to nourish and nurture a child with their milk, from the decisions about employment or social acceptance by individual women, to programs testing the cost and effectiveness of financial incentives for breastfeeding and identifying the financial and economic incentives for formula feeding, to evidence on the role of commercial marketing in changing breastfeeding practices and culture, to consideration of how the WHO/UNICEF *Global Strategy* for *Infant and Young Child Feeding* can be properly financed and implemented in both low- and high-income countries, to the benefit of the poor as the rich and women as well as men.

This special issue of the International Breastfeeding Journal catalyzes a focussed discussion on the economic influences on and consequences of breastfeeding, mothers’ milk, and premature weaning and how this links to redressing ‘the gender gap’, in the hope that exploration of this unusual topic will contribute to improved country and international policies and practices, such as paid maternity leave and appropriate maternal and child health care, that better enable all women to be supported to breastfeed as is optimal to their needs and situation.
